# Frequency-based rare diagnoses as a novel and accessible approach for studying rare diseases in large datasets: a cross-sectional study

**DOI:** 10.1186/s12874-023-01972-y

**Published:** 2023-06-17

**Authors:** Thomas S. Tröster, Viktor von Wyl, Patrick E. Beeler, Holger Dressel

**Affiliations:** 1 Division of Occupational and Environmental Medicine, Epidemiology, Biostatistics and Prevention Institute, University of Zurich and University Hospital Zurich, Zurich, Switzerland; 2 Epidemiology, Biostatistics and Prevention Institute, University of Zurich, Zurich, Switzerland; 3 Institute for Implementation Science in Health Care, University of Zurich, Zurich, Switzerland; 4 Center for Primary and Community Care, University of Lucerne, Lucerne, Switzerland

**Keywords:** Rare disease [MAJR], Rare diseases/epidemiology [MeSH Terms], Rare diseases/mortality [MeSH Terms], Rare diseases/statistics and numerical data [MeSH Terms]

## Abstract

**Background:**

Up to 8% of the general population have a rare disease, however, for lack of ICD-10 codes for many rare diseases, this population cannot be generically identified in large medical datasets. We aimed to explore *frequency-based rare diagnoses* (FB-RDx) as a novel method exploring rare diseases by comparing characteristics and outcomes of inpatient populations with FB-RDx to those with rare diseases based on a previously published reference list.

**Methods:**

Retrospective, cross-sectional, nationwide, multicenter study including 830,114 adult inpatients. We used the national inpatient cohort dataset of the year 2018 provided by the Swiss Federal Statistical Office, which routinely collects data from all inpatients treated in any Swiss hospital. Exposure: FB-RDx, according to 10% of inpatients with the least frequent diagnoses (i.e.1.decile) vs. those with more frequent diagnoses (deciles 2–10). Results were compared to patients having 1 of 628 ICD-10 coded rare diseases. Primary outcome: In-hospital death. Secondary outcomes: 30-day readmission, admission to intensive care unit (ICU), length of stay, and ICU length of stay. Multivariable regression analyzed associations of FB-RDx and rare diseases with these outcomes.

**Results:**

464,968 (56%) of patients were female, median age was 59 years (IQR: 40–74). Compared with patients in deciles 2–10, patients in the 1. were at increased risk of in-hospital death (OR 1.44; 95% CI: 1.38, 1.50), 30-day readmission (OR 1.29; 95% CI 1.25, 1.34), ICU admission (OR 1.50; 95% CI 1.46, 1.54), increased length of stay (Exp(B) 1.03; 95% CI 1.03, 1.04) and ICU length of stay (1.15; 95% CI 1.12, 1.18). ICD-10 based rare diseases groups showed similar results: in-hospital death (OR 1.82; 95% CI 1.75, 1.89), 30-day readmission (OR 1.37; 95% CI 1.32, 1.42), ICU admission (OR 1.40; 95% CI 1.36, 1.44) and increased length of stay (OR 1.07; 95% CI 1.07, 1.08) and ICU length of stay (OR 1.19; 95% CI 1.16, 1.22).

**Conclusion(s):**

This study suggests that FB-RDx may not only act as a surrogate for rare diseases but may also help to identify patients with rare disease more comprehensively. FB-RDx associate with in-hospital death, 30-day readmission, intensive care unit admission, and increased length of stay and intensive care unit length of stay, as has been reported for rare diseases.

**Supplementary Information:**

The online version contains supplementary material available at 10.1186/s12874-023-01972-y.

## Background

Rare diseases (RD) are a heterogeneous group of disorders concerning a broad range of medical specialties [1]. It has been estimated that 3.5–5.9% of the general population are affected by RD [2] and a similar study found a cumulative prevalence of 6.2%^3^, which is in line with the 6–8% suggested by the council of the European union [4]. Yet these numbers differ from the 0.33-2% estimated based on registries and inpatient data [5–9].

Moreover, identifying RD patients is a major obstacle. A first challenge is the lack of comprehensive rare disease registries. For example, Orphanet, an international endeavor to collect information on all RD, lists 753 registries [10]. Nevertheless, these registries are often limited in the number of diseases or regions they cover. Few countries, such as Italy in 2001 [11] and France in 2007 [12], managed to implement a general registry recording all known RD, which could be considered a desirable gold standard for RD research.

A further challenge pertains to the definition of rare disease classifications in electronic health records. For example, Orphanet provides an open access RD classification scheme based on unique ORPHACodes [13]. Supported by the European Commission Expert Group on RD recommendations [14] and the Europaen funded RD-Code project [15], multiple European countries have set to implement ORPHAcodes in routine coding systems [16, 17].

If no such system is in place, ICD-10 code references can be used. Walker et al. mapped 585 ORPHACodes to 1,084 ICD-10 codes, thereby mapping a total of 468 distinct RD to ICD-10 codes. However, the “RD resource set“ by Walker et al. [5] excluded infectious diseases. Similarly, OrphaData, Orphanet’s open data platform, links 6,847 ORPHACodes with 2,064 ICD-10 codes [18]. However, these ICD-10 based RD definitions have significant limitations. Complex RD may have no ICD-10 code at all, for instance, the 2015 ICD-10 version only counts 355 specific codes for RD [16]. Some ICD-10 codes may refer to multiple ICD-10 codes at once, while some assigned ICD-10 codes are often also used for more common diagnoses such as “Maturity onset diabetes of the young “ and “Type 2 diabetes mellitus without complications” [9]. In short, “Rare diseases and cross-referencing” by Orphanet [13] too maps numerous RD to ICD-codes of non-rare diseases. Recently, Blazsik and Beeler et al., proposed an improved and extended catalog of ICD-10 coded RD that combines [9] ICD-10 based RD with frequency-based RD definition. Their analysis was based on a large, single-center hospital database and may therefore have limited generalizability. The 11th revision of the ICD will offer numerous improvements in that context, e.g. providing ten times more specific RD codes than ICD-10. Unfortunately, the revision process was plagued by hurdles and setbacks, and still only about half of the currently 9’370 on Orphanet listed clinical entities known today are covered [16, 18], while the number of known RD still increases by approximately 100 newly discovered RD per year [1, 16].

Switzerland has implemented a national concept for rare diseases, which was adopted in 2014 [17, 19]. The concept aims to improve access to diagnoses and therapies, support patients, promote international research, and enhance clinical documentation and education for rare diseases. As part of the concept, national reference centers have been established, and a national registry for rare diseases was inititated in 2013, which was approved by the ethics committee in 2018 [20]. The registry uses ORPHACodes for data acquisition [21]. Professional coders in Switzerland code inpatient diagnoses using ICD10-GM. But there is currently no mandatory requirement to use ORPHAcodes in patients with (suspected) rare disease or report them to a registry.

Therefore, the question remains, whether generalizable, *frequency-based* RD definitions are feasible and comparable to catalogue-based RD systems, both with respect to included diagnoses as well as to health outcomes such as rehospitalization and mortality. By using a complete, nationwide dataset of hospital diagnoses, the present study aims (i) to suggest and investigate *frequency-based rare diagnoses* (FB-RDx) as an alternative to study a broad range of rare conditions and (ii) to investigate whether FB-RDx are associated with worse clinical outcomes.

## Methods

### Study design

This was a retrospective cross-sectional study. We used the national inpatient cohort dataset of the year 2018 provided by the Swiss Federal Statistical Office [22], which routinely collects data from all inpatients treated in any Swiss hospital. The dataset includes more than 700 variables, among them: demographic data (age at admission, sex, citizenship [Swiss vs. non-Swiss], type of insurance), administrative data (type of hospital [e.g. tertiary care academic medical center]), information on where the patient was admitted from [e.g. home], discharge destination [e.g. retirement home], type of admission [emergency vs. planned], clinical information [e.g. up to 50 ICD-10 coded diagnoses per person] and information on outcomes (in-hospital mortality, LOS, LOS in intensive care unit [ICU], number of days until readmission to the same or a different hospital).

In this study, ICD-10 codes were truncated to four digits for compatibility with the official ICD-10 code catalog as published by the World Health Organization [23].

The present study used completely anonymous data and conformed with the local law and the ethical review and research policies. Our study adhered to the STrengthening the Reporting of OBservational studies in Epidemiology (STROBE) guidelines [22, 24].

### Setting

In 2018, Switzerland’s hospital-related health care system consisted of 38,051 beds in 281 hospitals, and patients had a total of 1,443,626 hospital stays [25]. Switzerland uses the ICD-10-GM diagnosis coding system (GM: German Modification, DIMDI, Cologne, Germany). In the studied period the 2016 Swiss adaptation of the ICD-10-GM was used.

### Participants and study period

We included all adult (aged ≥ 18 at admission) inpatients who had at least one hospital stay with one or more diagnoses. All patients were discharged between 1st of January and 31st of December 2018.

We randomly selected only one stay for each included patient: On the one hand, when a patient has repeated stays for the same condition, some codes may be overrepresented; on the other hand, codes for the same condition may change due to improved diagnostic assessment over multiple stays, resulting in underrepresentation of the correct codes. Therefore, to avoid bias and to prevent such prevalence errors from patients with multiple stays, we only considered one random stay per patient.

### Primary and secondary outcomes

The primary outcome was the association of FB-RDx with in-hospital death.

Secondary outcomes were: Associations of FB-RDx with LOS, 30-day readmissions, admissions to an ICU, and ICU LOS. We compared all results to associations of RD with the same outcomes.

### Predictors

Primary predictor was having FB-RDx. Models were also run for the presence of a RD.

#### Frequency-based rare diagnosis definition

The frequency of all diagnoses in our dataset was used to determine whether a diagnosis was rare. Patients were grouped into ten quantiles, i.e. deciles, based on each patient’s least frequent diagnosis in this dataset. Therefore, the first decile included the 10% of patients with the rarest diagnoses in this dataset, in contrast to the tenth decile, in which the 10% of patients were found whose rarest diagnosis was still among the most frequent diagnoses.

A post-hoc exploratory sensitivity analysis added models using 20% and 30% as an alternative lowest quantile as well as whether excluding ICD-Codes not associated with diseases (Chaps. 18–22: “XVIII Symptoms, signs and abnormal clinical and laboratory findings, not elsewhere classified”, “XIX Injury, poisoning and certain other consequences of external causes”, “XX External causes of morbidity and mortality”, “XXI Factors influencing health status and contact with health services”, “XXII Codes for special purposes”) would substantially affect our results.

#### ICD-10 based rare disease definition (RD)

Having an RD was defined according to an ICD-10 coded RD reference catalog by Blazsik and Beeler [9]. A supplementary analysis used presence of a diagnoses in “RD resource set” by Walker et al. [5] as predictor. [Additional file 1: eTable1] A table with all ICD-10 codes in our dataset, their frequencies, their decile groups and whether they are a part of the Blazsik and Beeler et al. [9], Walker et al. [5] or the OrphaData catalog [13] has been provided in the online supplementary. [Additional file 2]

### Co-Variables

All models adjusted for age, sex, admission from home, Swiss citizenship, number of diagnoses (excluding the rarest diagnosis), type of admission and class of insurance (mandatory insurance only, supplementary hospital insurance [semi-privat or privat]). Analysis of the outcome 30-day readmission additionally adjusted for length of stay. Models for LOS, ICU-LOS and 30-day readmissions excluded patients who died during the stay, and the models for ICU admissions, LOS, ICU-LOS and 30-day readmissions excluded rehabilitation clinics. In all models analyzing FB-RDx and RD, the variable number of diagnoses excluded FB-RDx and ICD-10 coded RDs, respectively. To compensate for potentially non-linear effects, we used restricted cubic splines [26] for the variable age. This allows for non-linear adjustment but reduces its interpretability of the effect size of age. Further explanation has been provided by Gauthier et al. [27] However, to demonstrate effect size and comparability, we additionally included an analysis using categorical age-groups. [Additional file 1: eTables 2 and 3]

### Statistical analysis

Non-normal distributed variables are presented as medians with interquartile ranges (IQR), categorical variables are presented as counts with percentages. Chi-square tests were used to compare categorical variables, Kruskal-Wallis tests to compare continuous variables between groups.

To transform skewed outcomes, we log-transformed LOS and ICU LOS, thereby allowing the application of linear regression, as described elsewhere [28]. Multivariable regression was performed with all outcomes using both FB-RDx and RD as predictor modalities.

Statistical analyses were computed with R, version 3.6.2 (R Foundation for Statistical Computing, Vienna, Austria). Calculations for restricted cubic splines and baseline characteristics were performed using the “rms” and “tableone” packages, respectively.

## Results

**Fig. 1:**
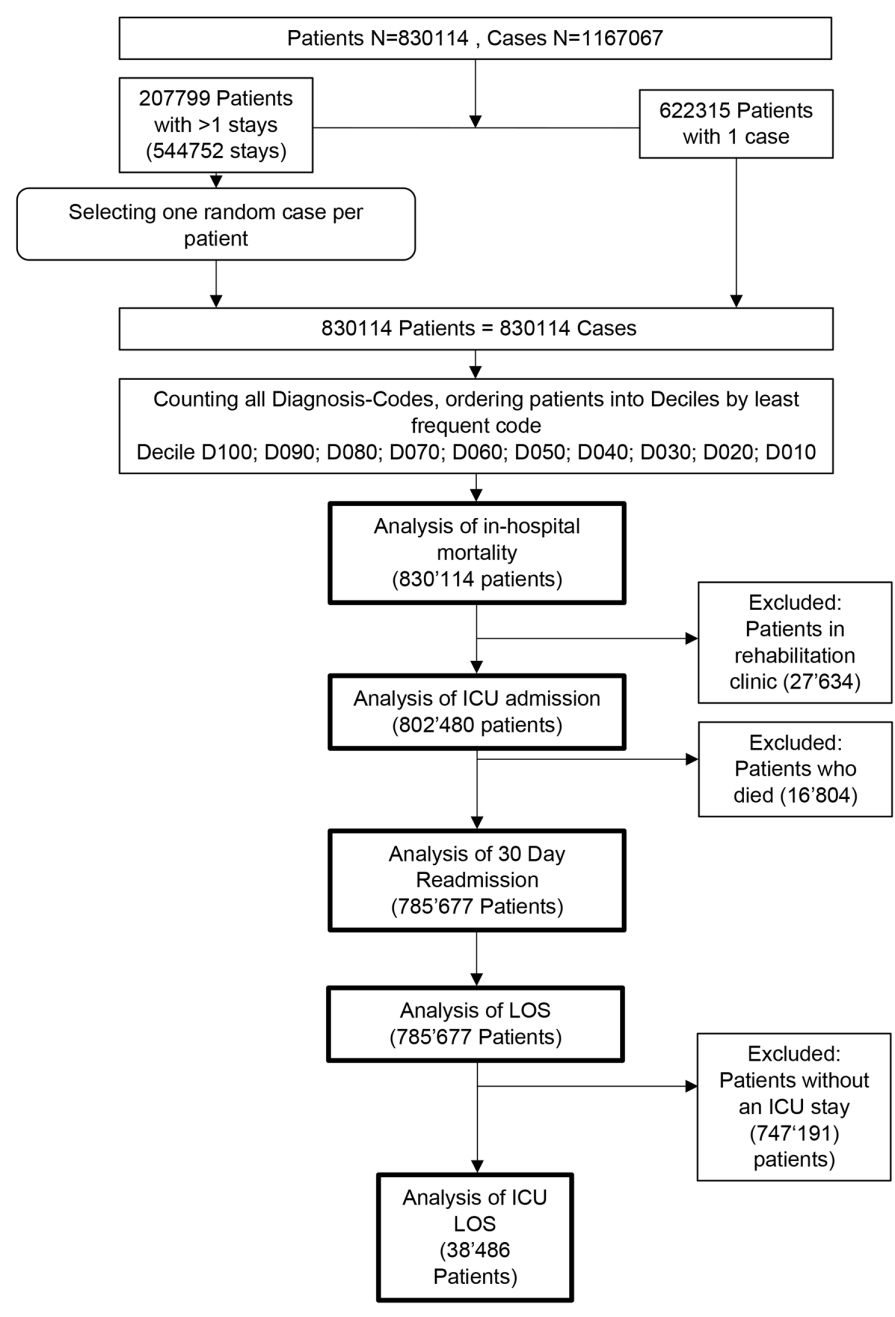
Patient flow diagram and performed outcome analyses

Overall, 830,114 patients with a total of 1,167,067 stays were considered in our study. 622,315 (75.0%) patients had one stay, and 207,799 (25.0%) patients with more than one stay subsumed a total of 544,752 stays (average 1.41 stays per patient). After randomly selecting a single stay per patient, 830,114 patients with one stay each were included (Fig. 1). A total of 7,643 distinct ICD-10 codes were identified. Table 1 illustrates the baseline characteristics stratified by decile groups.

**Table 1:** Baseline characteristics stratified by deciles (rarest diagnoses in 1. decile)

	Overall		1. decile	2.-9. decile	10. decile
Number of patients per group	830,114		83,720	664,236	82,158
age (median [IQR])	59.00 [40.00, 74.00]		59.00 [40.00, 74.00]	60.00 [41.00, 75.00]	56.00 [35.00, 70.00]
Age group % (freq)					
18–34	18.4 (152,415)		18.4 (15,442)	17.6 (116,880)	24.5 (20,093)
35–49	17.1 (142,208)		17.6 (14,773)	17.1 (113,587)	16.9 (13,848)
50–64	22.5 (186,912)		22.7 (19,016)	22.4 (148,850)	23.2 (19,046)
65–79	25.6 (212,848)		25.5 (21,316)	25.5 (169,558)	26.7 (21,974)
80 and older	16.4 (135,731)		15.7 (13,173)	17.4 (115,361)	8.8 (7197)
Sex = F % (freq)	56.0 (464,968)		53.2 (44,561)	56.6 (375,659)	54.5 (44,748)
Numbers of stays % (freq)					
1	75.0 (622,315)		67.0 (56,133)	74.7 (496,149)	85.2 (70,033)
2	16.4 (136,089)		19.2 (16,089)	16.6 (110,498)	11.6 (9502)
3	5.0 (41,779)		7.2 (6050)	5.1 (33,916)	2.2 (1813)
4	2.0 (16,205)		3.2 (2670)	2.0 (12,996)	0.7 (539)
>4	1.7 (13,726)		3.3 (2778)	1.6 (10,677)	0.3 (271)
Number of diagnoses per stay (median [IQR])	6.00 [3.00, 9.00]		8.00 [5.00, 12.00]	6.00 [3.00, 9.00]	3.00 [1.00, 5.00]
Insurance class % (freq)					
Mandatory insurance	75.7 (628,134)		78.2 (65,491)	75.6 (502,095)	73.7 (60,548)
Semi-private insurance	15.0 (124,827)		13.4 (11,200)	15.1 (100,075)	16.5 (13,552)
Private insurance	9.3 (77,153)		8.4 (7029)	9.3 (62,066)	9.8 (8058)
Swiss citizenship % (freq)	78.5 (651,955)		76.7 (64,229)	78.8 (523,293)	78.4 (64,433)
Admission from home % (freq)	90.4 (750,314)		86.0 (72,026)	90.3 (599,518)	95.9 (78,770)
Type of admission % (freq)					
Emergency	44.5 (369,162)		49.9 (41,754)	45.7 (303,403)	29.2 (24,005)
Planned	53.7 (445,947)		47.4 (39,669)	52.5 (348,718)	70.1 (57,560)
Other	1.8 (15,005)		2.7 (2297)	1.8 (12,115)	0.7 (593)
Hospital category admitted to % (freq)					
Tier 1 University hospital	14.5 (120,186)		25.3 (21,201)	13.9 (92,066)	8.4 (6919)
Tier 2 Center hospital	53.3 (442,732)		48.7 (40,751)	54.2 (359,972)	51.1 (42,009)
Tier 3 General hospital	8.6 (71,541)		6.7 (5576)	8.7 (57,687)	10.1 (8278)
Tier 4 General hospital	10.0 (83,400)		7.0 (5849)	10.0 (66,524)	13.4 (11,027)
Tier 5 General hospital	1.3 (10,777)		1.1 (926)	1.3 (8848)	1.2 (1003)
Other speciality	1.7 (14,433)		2.3 (1939)	1.7 (11,338)	1.4 (1156)
Rehabilitation clinic	3.3 (27,634)		5.3 (4401)	3.2 (21,289)	2.4 (1944)
Surgical clinic	7.2 (59,411)		3.7 (3077)	7.0 (46,512)	12.0 (9822)
Discharged to % (freq)					
Deceased	2.0 (16,921)		3.8 (3206)	2.0 (13,575)	0.2 (140)
Home	86.1 (715,099)		78.9 (66,050)	86.0 (571,359)	94.6 (77,690)
Nursing Home	2.8 (22,924)		3.5 (2942)	2.9 (19,570)	0.5 (412)
Retirement Home	1.6 (13,181)		1.9 (1566)	1.7 (11,357)	0.3 (258)
Psychiatry	1.3 (10,438)		3.0 (2547)	1.2 (7680)	0.3 (211)
Rehabilitation	3.4 (28,142)		4.9 (4073)	3.3 (22,050)	2.5 (2019)
Acute Hospital	2.2 (18,524)		3.1 (2567)	2.2 (14,871)	1.3 (1086)
Other	0.6 (4885)		0.9 (769)	0.6 (3774)	0.4 (342)
Number of RD (mean (SD))	0.08 (0.29)		0.23 (0.50)	0.07 (0.27)	0.00 (0.00)
Has a RD % (freq)	7.2 (59,861)		19.6 (16,441)	6.5 (43,420)	0.0 (0)
Outcomes					
Died in hospital % (freq)	2.0 (16,921)		3.8 (3206)	2.0 (13,575)	0.2 (140)
LOS (median [IQR])	4.00 [3.00, 7.00]		6.00 [3.00, 11.00]	4.00 [3.00, 7.00]	4.00 [3.00, 6.00]
30-day Readmission % (freq)	3.9 (32,698)		6.1 (5082)	4.0 (26,354)	1.5 (1262)
Stayed in ICU % (freq)	5.2 (43,171)		10.5 (8811)	5.0 (32,943)	1.7 (1417)
Of those in ICU (N = 43‘171)					
Hours in ICU (median [IQR])	25.00 [18.00, 55.00]		38.00 [20.00, 90.00]	25.00 [18.00, 49.00]	21.00 [15.00, 26.00]

### Primary end point

#### Frequency-based rare diagnoses (FB-RDx)

Unadjusted logistic regression indicated that FB-RDx associate with increased in-hospital mortality (patients in the first decile: odds ratio [OR] 2.13; 95% confidence interval [CI]: 2.05 to 2.21). Multivariable logistic regression showed an independent association of FB-RDx with increased in-hospital mortality (1st Decile vs. 2nd -10th: OR 1.44; 95% CI: 1.38 to 1.50) as shown in Table 2.

**Table 2:** In-hospital mortality logistic-regression models

In-Hospital mortality in Deciles		
Unadjusted model		
Variable	OR	(95% CI)
1st Decile (vs. 2nd -10th Deciles)^a^	2.13	(2.05,2.21)
**Adjusted model**		
Variable	OR	(95% CI)
1st Decile (vs. 2nd -10th Deciles)^a^	1.44	(1.38,1.50)
Female sex	0.73	(0.70,0.75)
Swiss citizen	1.02	(0.97,1.07)
Admissioned from home	0.63	(0.60,0.66)
Age Group^**b**^ (vs. Age Group 1)		
Age Group 2	1.09	(1.07,1.11)
Age Group 3	1.03	(0.96,1.11)
Age Group 4	0.72	(0.60,0.87)
Age Group 5	2.19	(1.79,2.68)
Nr of Diagnoses (vs. 0 non-rare Diagnose)^c^		
1 Diagnoses	0.34	(0.21,0.53)
2 Diagnoses	0.99	(0.59,1.66)
3 Diagnoses	0.32	(0.20,0.49)
5 Diagnoses	1.22	(0.76,1.98)
6 Diagnoses	0.43	(0.27,0.67)
7 Diagnoses	0.56	(0.36,0.87)
8 Diagnoses	0.67	(0.43,1.03)
9 Diagnoses	0.86	(0.55,1.33)
10 Diagnoses	0.99	(0.63,1.53)
11–12 Diagnoses	1.24	(0.80,1.92)
13–15 Diagnoses	1.49	(0.96,2.31)
>=16 Diagnoses	1.96	(1.26,3.03)
Admission type (vs. Emergency)		
Elective	0.36	(0.35,0.38)
other	0.46	(0.42,0.50)
Insurance class (vs. general)		
semiprivate	0.86	(0.82,0.90)
private	0.86	(0.81,0.91)
Hospital Category/Size (vs. N1- University Hospital)		
Tier 2 Center hospital	0.86	(0.82,0.90)
Tier 3 General hospital	0.69	(0.65,0.74)
Tier 4 General hospital	0.78	(0.72,0.84)
Tier 5 General hospital	1.21	(1.07,1.36)
Other speciality	1.65	(1.49,1.83)
Rehabilitation clinic	0.2	(0.16,0.24)
Surgical clinic	0.15	(0.11,0.19)

^a^«D010»=10% of patients with the rarest diseases. «D100 »=10% of the patients with the most common diseases; ^b^Age was grouped using restricted cubic splines; ^c^Rare Diagnoses leading to an 1st -Decile were subtracted;

**Fig. 2:**
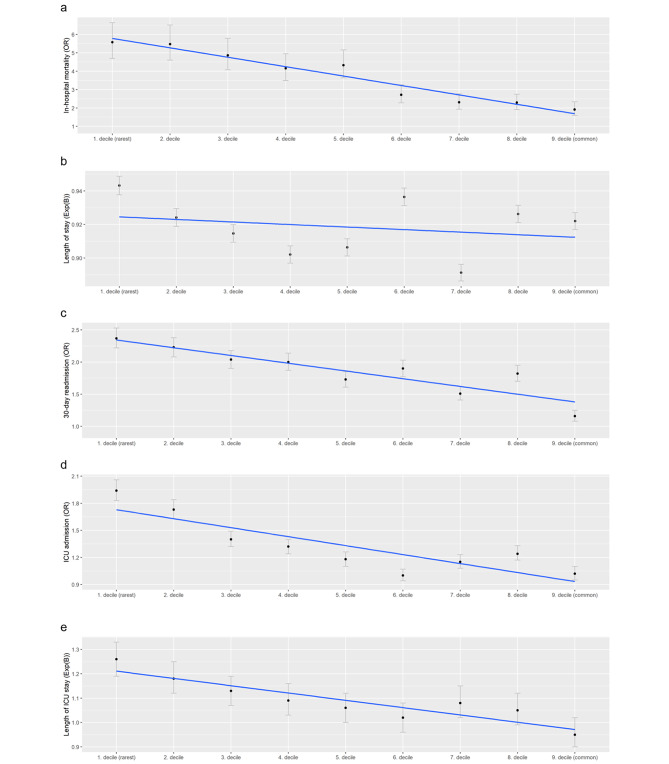
Dose-response relationship between outcomes and frequency-based rare diagnoses; Fig. 2a-e illustrates the dose-response relationship between our outcomes, frequency-based rare diagnoses. Except for LOS, rarer diagnoses where significantly associated with worse clinical outcomes, highlighted by the fitted linear model (blue line). Fig. 2**a** In-hospital mortality. Fig. 2**b** Length of stay. Fig. 2**c** 30-day readmission. Fig. 2**d** ICU admission. Fig. 2**e** Length of ICU stay

Stratified by all ten deciles, the first decile (*rarest*) demonstrated the strongest association (1st Decile: OR 5.58; CI: 4.69 to 6.64), subsequently decreasing with each more common decile (9th Decile: OR 1.92; CI: 1.59 to 2.33) on a linear slope (Fig. 2a).

#### Rare diseases (RD)

Having an RD and being in the rarest decile had comparable associations with increased in-hospital mortality (RD: OR 1.82; CI: 1.75 to 1.89) (Table 3).

**Table 3:** Regression models comparing associations of FB-RDx (first decile) with five outcomes vs. having a rare disease

In-hospital mortality						
Predictor	OR	(95% CI)		Predictor	OR	(95% CI)
First decile	1.44	(1.38,1.50)		RD	1.82	(1.75,1.89)
**LOS** ^**a**^						
**Predictor**	**Exp(B)**	**(95% CI)**		**Predictor**	**Exp(B)**	**(95% CI)**
First decile	1.03	(1.03,1.04)		RD	1.07	(1.07,1.08)
**30-day readmission** ^**a,b**^						
**Predictor**	**OR**	**(95% CI)**		**Predictor**	**OR**	**(95% CI)**
First decile	1.29	(1.25,1.34)		RD	1.37	(1.32,1.42)
**ICU admission**						
**Predictor**	**OR**	**(95% CI)**		**Predictor**	**OR**	**(95% CI)**
First decile	1.50	(1.46,1.54)		RD	1.40	(1.36,1.44)
**ICU LOS** ^**a, c**^						
**Predictor**	**Exp(B)**	**(95% CI)**		**Predictor**	**Exp(B)**	**(95% CI)**
First decile	1.15	(1.12,1.18)		RD	1.19	(1.16,1.22)

All models are adjusted for sex, swiss citizenship, admission from home, age group (using restricted cubic splines), Nr of Diagnoses, Admission type, Insurance class and hospital class; Nr of Diagnoses excluded Diagnoses in the first decile or the Blazsik/Beeler-Reference respectively; ^a^ LOS, 30-Day Readmission and ICU-LOS excluded patients who deceased during their stay; ^b^ 30-day readmission was additionally adjusted for LOS; ^c^ ICU-LOS only included patients being admitted to an ICU.

Abbreviations: LOS = length of stay; ICU = intensive care unit; OR = odds ratio;

### Secondary end points

#### Frequency-based rare diagnoses (FB-RDx)

FB-RDx were independently associated (1st Decile vs. 2nd -10th) with increased LOS (OR 1.03; CI: 1.03 to 1.04), 30-day readmissions (OR 1.29; CI: 1.25 to 1.34), ICU admissions (OR 1.50; CI: 1.46 to 1.54) and increased ICU LOS (OR 1.15; CI: 1.12 to 1.18). Except for LOS, all outcomes demonstrated a dose-effect relationship with lower (*rarer*) deciles having a larger impact. (Fig. 2b-e)

#### Rare diseases (RD)

RD were associated with 30-days readmissions, increased LOS, ICU admissions and increased ICU LOS with comparable effect sizes to those of FB-RDx (Table 3).

#### Post-hoc supplementary analysis

A post-hoc exploratory sensitivity analysis on larger quantiles demonstrated a stronger association on in-hospital mortality ([1st -2nd Deciles vs. 3rd -10th Deciles: OR 1.57; CI: 1.52 to 1.63] and [1st -3rd Deciles vs. 4th -10th Deciles: OR 1.76; CI: 1.70 to 1.83]) as well as on the secondary outcomes.

To further investigate the effect of Deciles on LOS, a subgroup analysis stratified by hospital-class (Tier 1- University hospital vs. others) was added. These models showed an association of increased LOS and FB-RDx in patients hospitalized in a university hospital, while patients in lower-tier hospitals showed no such effect (not shown). A sensitivity-analysis excluding ICD-Chapters not associated with rare disease (Chapters XVIII-XXII) was performed. For 1st Decile vs. 2nd -10th, this resulted in OR 1.42 (95% CI: 1.36 to 1.48), stratified by Decile in 1st Decile: OR 4.51 (CI: 4.0 to 5.08) and 9th Decile: OR 1.53 (CI: 1.32 to 1.78) and for having an RD in OR 1.52 (CI: 1.46 to 1.59) (not shown).

## Discussion

This study used administrative healthcare data to test FB-RDx as an approach to identify patients with rare conditions similar to RD. Our analysis shows that FB-RDx are independently associated with worse inpatient outcomes in respect of in-hospital mortality, increased LOS, 30-day readmissions, ICU admissions, and increased ICU LOS. We also demonstrated an independent dose-effect relationship between deciles and in-hospital mortality, 30-day readmissions, ICU admissions, and ICU LOS, but not for LOS (Fig. 2a-e). This suggests a linear association between rarity of diagnoses and worse clinical outcomes, where *rarer diagnoses* are associated with worse outcomes.

There is few reported data on RD inpatient-outcomes. A pediatric study found a higher in-hospital mortality rate and an increased LOS in children hospitalized in relation to birth defects and genetic diseases [29]. An Italian RD registry reported a raw annual mortality rate of 13.0/100,000 among patients with RD [8]. Regarding increased 30-day readmission rates in patients with FB-RDx, our findings were comparable to a previous study analyzing muscular dystrophies, spina bifida and fragile X syndrome (OR 3.61 to 5.67) [30].

Although we were unable to find adjusted analyses, some research groups suggested that LOS is increased among inpatients with RD. Chiu et al. reported a marginal increase of the LOS by 0.3 days to a LOS of 6.1 days in patients with RDs [6]. Walker et al. reported an increase from 3.8 days (patients without RDs) to 5.5 days (patients with RDs) in Western Australia [5]. In our study, RD were also associated with an increased LOS, however, FB-RDx were only marginally associated when comparing the patients in the first decile (with the *rarest diagnoses*) to the other 90% of patients. Explorative subgroup analysis showed an association of increased LOS and FB-RDx in patients hospitalized in a university hospital, while patients in lower-tier hospitals showed no such effect. We suspect this being due to hospitals referring these patients to more specialized clinics for further, and often prolonged, diagnostics and treatments.

To our knowledge, this is the first study using FB-RDx as a novel approach to investigate patients with a broad range of rare conditions similar to RD. Compared to previous work [5–7, 9] the present approach could be considered the most comprehensive approach for the purpose of identifying RD-characteristic patients, since no registry, catalog or otherwise limited resource is needed to find the patients of interest. Further strengths of this multicenter study were: We investigated several clinically important and generalizable inpatient outcomes with economic implications, we used a nationwide dataset, and we compared FB-RDx with the most comprehensive ICD-10 coded catalog of RD currently available [9]. As we included all adult inpatients staying at Swiss hospitals, we analyzed diverse patient populations treated in clinical units that cover all medical specialties for adults. Our models were adjusted for various potentially important co-variables. However, several weaknesses should be considered in interpreting our study. First, the study period was limited to one year, however, we still included over 800,000 patients. Second, this study does not represent the general population, as only inpatients were included in our dataset. Third, we only included patients aged ≥ 18 years, whereas approximately 23% of patients with RD are < 18 years old [8]. Fourth, many ICD chapters are unlikely to be associated with rare disease (e.g. Chapter XX External causes of morbidity and mortality), or are associated with high mortality RD (e.g. Chapter II Neoplasms), possibly distorting the data. We performed an exploratory post-hoc sensitivity analysis excluding ICD chapters not associated with rare diseases (Chapters XVIII-XXII), which did not produce a substantial change in results. Lastly, rare diseases are underrecognised and underreported, a bias that may be minimized by increased awareness among healthcare professionals [31]. Therefore, healthcare professionals should be sensitized and educated more on this issue early on.

Due to the inherent scarcity of data on RD and limited RD registries, methods should be developed to exhaust the utilization, application and interpretation of large data sources such as administrative hospital data. For example, in one study, researchers developed an electronic phenotyping algorithm to increase the detection rate of Becker and Duchenne muscular dystrophy among patients with the broader ICD-9 code 359.1 (hereditary progressive muscular dystrophy) and were thereby able to further study the clinical outcomes of those muscular dystrophies [32]. Similar to our automated method, such approaches have to be refined, but promise improved epidemiological research capabilities for RD in settings where more comprehensive data like large-scale registries are not yet available.

Our study suggests that FB-RDx may be another novel way to analyze this heterogeneous and otherwise difficult to identify population. Given the absence of more precise identification methods (like general registries and widespread ORPHACode implementation, as discussed in the introduction), and due to wide-spread use of ICD coding, FB-RDx provide a means to demonstrate the impact of rare diseases on healthcare systems. This may help to raise awareness and garner support for this vulnerable population. To approximate this RD population in our *rare diagnosis* approach, we decided to use deciles. This is based on previous works on inpatients: Walker et al. reported that 4.6% of all discharged inpatients have an RD identified by a catalog of 468 ICD-10 codes [5]. Using the same resource, we identified 4.7% of our inpatients suffering from an RD. However, as ICD10-AM codes were not specificly translated into ICD10-GM codes, discrapencies cannot be ruled out. A previous study at a Swiss university hospital found 11.5% (7.2% in our study population) of the inpatients having an RD identified using the extended ICD-10 code catalog [9]. The proportion of RD across the deciles decreased from nearly 20% in the first to 0% in the tenth decile.

However, the question of which percentile of the population, those with the rarest ICD-10 codes, is the best quantile to capture patients with RD, needs to be addressed in future work. An exploratory post-hoc sensitivity analysis suggested larger quantiles, e.g. 20% and 30% cut-offs for future starting points.

In conclusion, this study used FB-RDx, defined by the frequency of diagnoses in administrative hospital data, as a novel approach to comprehensively identify patients with rare conditions similar to RD. FB-RDx are independently associated with in-hospital mortality, ICU admissions, increased ICU LOS, and 30-day readmissions, as were RD.

## Electronic supplementary material

Below is the link to the electronic supplementary material.

Supplementary Material 1

Supplementary Material 2

## Data Availability

The data that support the findings of this study are available from the Swiss Federal Statistical Office but restrictions apply to the availability of these data, which were used under license for the current study, and so are not publicly available. However, data are available from the Swiss Federal Statistical Office upon filing an application with a study proposal, and after signing a data protection contract [22]. (E-Mail contact: Gesundheit_DSV@bfs.admin.ch)

## Abbreviations

CI: Confidence interval
FB-RDx: Frequency-based rare diagnoses
ICU: Intensive care unit
IQR: Interquartile range
LOS: Length of stay
OR: Odds ratio
RD: Rare disease
SQL: Structured query language
