# Supplementary material for: Frequency-based rare diagnoses as a novel and accessible approach for studying rare diseases in large datasets: a cross-sectional study
Source: BMC Med Res Methodol. 2023 Jun 17;23:143. doi: 10.1186/s12874-023-01972-y (PMC10276905; doi:10.1186/s12874-023-01972-y)
Supplement: Supplementary file 1 — Supplementary Material 1 [file 12874_2023_1972_MOESM1_ESM.docx]

| **eTable 1 Logistic regression models* for the effect of having a Disease in “RD resource set“ by Walker et al.** | | |
| --- | --- | --- |
| **In-Hospital Mortality** | | |
| Variable | OR | (95% CI) |
| Has Walker-Reference | 1.64 | (1.56,1.73) |
|  |  |  |
| **LOS^a^** | | |
| Variable | Exp(B) | (95% CI) |
| Has Walker-Reference | 1.11 | (1.10,1.12) |
|  |  |  |
| **30 Day Readmission^a,b^** | | |
| Variable | OR | (95% CI) |
| Has Walker-Reference | 1.44 | (1.38,1.51) |
|  |  |  |
| **ICU Admission** | | |
| Variable | OR | (95% CI) |
| Has Walker-Reference | 1.42 | (1.37,1.48) |
|  |  |  |
| **ICU LOS^a,c^** | | |
| Variable | Exp(B) | (95% CI) |
| Has Walker-Reference | 1.12 | (1.09,1.16) |
| All models are adjusted for sex, swiss citizenship, admission from home, age group (using restricted cubic splines), Nr of Diagnoses, Admission type, Insurance class and hospital class; Nr of Diagnoses excluded Diagnoses in the “RD resource set“ by Walker et al.; ^a^ LOS, 30-Day Readmission and ICU-LOS excluded that deceased during their stay; ^b^ 30-day readmission was additionally adjusted for LOS; ^c^ ICU-LOS only included patients being admitted to an ICU. Abbreviations: LOS=length of stay; ICU=intensive care unit; OR=odds ratio; | | |

| **eTable 2: In-Hospital Mortality logistic-regression models on Deciles, adjusted for categorical age-groups** | | |
| --- | --- | --- |
| **In-Hospital mortality in Deciles** | | |
| **Unadjusted model** | | |
| Variable | OR | (95% CI) |
| Decile (vs Decile D100)^a^ |  |  |
| Decile D090 | 3.22 | (2.66,3.89) |
| Decile D080 | 5.84 | (4.88,6.99) |
| Decile D070 | 6.58 | (5.50,7.86) |
| Decile D060 | 8.46 | (7.10,10.08) |
| Decile D050 | 14.69 | (12.37,17.44) |
| Decile D040 | 16.38 | (13.81,19.44) |
| Decile D030 | 19.47 | (16.42,23.08) |
| Decile D020 | 23.85 | (20.13,28.26) |
| Decile D010 | 23.33 | (19.69,27.64) |
|  |  |  |
| **Adjusted model** | | |
| Variable | OR | (95% CI) |
| Decile (vs Decile D100)^a^ |  |  |
| Decile D090 | 1.94 | (1.60,2.35) |
| Decile D080 | 2.31 | (1.92,2.77) |
| Decile D070 | 2.32 | (1.94,2.78) |
| Decile D060 | 2.73 | (2.28,3.26) |
| Decile D050 | 4.31 | (3.62,5.14) |
| Decile D040 | 4.09 | (3.43,4.87) |
| Decile D030 | 4.77 | (4.01,5.69) |
| Decile D020 | 5.34 | (4.49,6.35) |
| Decile D010 | 5.39 | (4.52,6.41) |
|  |  |  |
| Female sex | 0.74 | (0.72,0.77) |
| Swiss citizen | 1.06 | (1.01,1.11) |
| Admissioned from home | 0.6 | (0.58,0.63) |
| Age Group (vs Age 18-34) |  |  |
| Age 35-49 | 4.25 | (3.48,5.19) |
| Age 50-64 | 12.37 | (10.30,14.87) |
| Age 65-79 | 19.34 | (16.13,23.19) |
| Age >=80 | 29.89 | (24.92,35.83) |
| Nr of Diagnoses (vs 1 Diagnose)^b^ |  |  |
| 3 Diagnoses | 0.85 | (0.74,0.97) |
| 5 Diagnoses | 1.03 | (0.91,1.17) |
| 6 Diagnoses | 1.21 | (1.07,1.37) |
| 7 Diagnoses | 1.46 | (1.29,1.64) |
| 8 Diagnoses | 1.78 | (1.58,2.00) |
| 9 Diagnoses | 2.07 | (1.84,2.34) |
| 10 Diagnoses | 2.47 | (2.19,2.78) |
| 11-12 Diagnoses | 2.92 | (2.61,3.27) |
| 13-15 Diagnoses | 3.73 | (3.34,4.16) |
| >=16 Diagnoses | 5.9 | (5.29,6.58) |
| Admission type (vs Emergency) |  |  |
| Elective | 0.36 | (0.34,0.37) |
| other | 0.45 | (0.42,0.50) |
| Insurance class (vs general) |  |  |
| semiprivate | 0.86 | (0.82,0.90) |
| private | 0.85 | (0.80,0.91) |
| Hospital Category/Size (vs N1- University Hospital) |  |  |
| Tier 2 Center hospital | 0.89 | (0.86,0.93) |
| Tier 3 General hospital | 0.73 | (0.68,0.78) |
| Tier 4 General hospital | 0.82 | (0.76,0.88) |
| Tier 5 General hospital | 1.26 | (1.12,1.43) |
| Other speciality | 1.68 | (1.52,1.86) |
| Rehabilitation clinic | 0.2 | (0.16,0.24) |
| Surgical clinic | 0.16 | (0.12,0.20) |
| ^a^«D010»=10% of patients with the rarest diseases. «D100 »=10% of the patients with the most common diseases;bNo patient with 2 diagnoses was recorded; ^b^Rare Diagnoses leading to an 1st-Decile were subtracted; | | |

| **eTable 3 Logistic regression models for hospital LOS, 30-Day Readmission, ICU-Admission and ICU-LOS adjusted for categorical age-groups instead of restricted cupic splines** | | | | | | | | | | | | | | |
| --- | --- | --- | --- | --- | --- | --- | --- | --- | --- | --- | --- | --- | --- | --- |
| **LOS**^a^ |  |  |  | **30 Day Readmission**^a,b^ |  |  |  | **ICU Admission** |  |  |  | **ICU LOS**^a, c^ |  |  |
| Variable | Exp(B) | (95% CI) |  | Variable | OR | (95% CI) |  | Variable | OR | (95% CI) |  | Variable | Exp(B) | (95% CI) |
| Decile (vs Decile D100) |  |  |  | Decile (vs Decile D100) |  |  |  | Decile (vs Decile D100) |  |  |  | Decile (vs Decile D100) |  |  |
| Decile D090 | 0.92 | (0.92,0.93) |  | Decile D090 | 1.16 | (1.07,1.25) |  | Decile D090 | 1.02 | (0.95,1.09) |  | Decile D090 | 0.95 | (0.89,1.02) |
| Decile D080 | 0.93 | (0.92,0.93) |  | Decile D080 | 1.81 | (1.69,1.94) |  | Decile D080 | 1.23 | (1.15,1.32) |  | Decile D080 | 1.06 | (1.00,1.12) |
| Decile D070 | 0.89 | (0.89,0.90) |  | Decile D070 | 1.5 | (1.40,1.61) |  | Decile D070 | 1.14 | (1.07,1.22) |  | Decile D070 | 1.09 | (1.02,1.15) |
| Decile D060 | 0.94 | (0.93,0.94) |  | Decile D060 | 1.89 | (1.76,2.02) |  | Decile D060 | 0.99 | (0.93,1.06) |  | Decile D060 | 1.02 | (0.96,1.08) |
| Decile D050 | 0.91 | (0.90,0.91) |  | Decile D050 | 1.72 | (1.61,1.84) |  | Decile D050 | 1.17 | (1.10,1.25) |  | Decile D050 | 1.06 | (1.00,1.12) |
| Decile D040 | 0.9 | (0.90,0.91) |  | Decile D040 | 1.99 | (1.86,2.13) |  | Decile D040 | 1.31 | (1.23,1.40) |  | Decile D040 | 1.1 | (1.04,1.16) |
| Decile D030 | 0.91 | (0.91,0.92) |  | Decile D030 | 2.03 | (1.89,2.17) |  | Decile D030 | 1.4 | (1.32,1.49) |  | Decile D030 | 1.13 | (1.07,1.20) |
| Decile D020 | 0.92 | (0.92,0.93) |  | Decile D020 | 2.22 | (2.07,2.37) |  | Decile D020 | 1.74 | (1.63,1.85) |  | Decile D020 | 1.19 | (1.12,1.25) |
| Decile D010 | 0.94 | (0.94,0.95) |  | Decile D010 | 2.36 | (2.21,2.53) |  | Decile D010 | 1.96 | (1.84,2.08) |  | Decile D010 | 1.26 | (1.20,1.33) |
| All models are adjusted for sex, swiss citizenship, admission from home, age group (categorical), Nr of Diagnoses, Admission type, Insurance class and hospital class; ^a^ LOS, 30-Day Readmission and ICU-LOS excluded that deceased during their stay; ^b^ 30-day readmission was additionally adjusted for LOS; ^c^ ICU-LOS only included patients being admitted to an ICU. Abbreviations: LOS=length of stay; ICU=intensive care unit; OR=odds ratio; | | | | | | | | | | | | | | |
